# Navigating Post-Operative Outcomes: A Comprehensive Reframing of an Original Graded Prognostic Assessment in Patients with Brain Metastases

**DOI:** 10.3390/cancers16020291

**Published:** 2024-01-10

**Authors:** Maria Goldberg, Michel G. Mondragon-Soto, Laura Dieringer, Ghaith Altawalbeh, Paul Pöser, Lea Baumgart, Benedikt Wiestler, Jens Gempt, Bernhard Meyer, Amir Kaywan Aftahy

**Affiliations:** 1 Department of Neurosurgery, School of Medicine, Klinikum Rechts der Isar, Technical University Munich, 80333 Munich, Germany; laura.dieringer@tum.de (L.D.); ghaith.altawalbeh@mri.tum.de (G.A.); bernhard.meyer@tum.de (B.M.); kaywan.aftahy@tum.de (A.K.A.); 2 Department of Neurosurgery, National Institute of Neurology and Neurosurgery, Mexico City 14269, Mexico; mmondragon@innn.edu.mx; 3 Department of Neurosurgery, Charite–Universitätsmedizin Berlin, Corporate Member of Freie Universität Berlin, Humboldt-Universität zu Berlin, and Berlin Institute of Health, 10117 Berlin, Germany; 4 Department of Neurosurgery, University Medical Center Hamburg-Eppendorf, 20246 Hamburg, Germany; 5 Department of Neuroradiology, School of Medicine, Klinikum Rechts der Isar, Technical University Munich, 80333 Munich, Germany; benedikt.wiestler@mri.tum.de

**Keywords:** GPA, brain metastasis, prognostic assessment, rest tumor volume, surgery

## Abstract

**Simple Summary:**

Prognostic evaluation in patients with advanced cancer is essential, as life expectancy influences important personal, as well as clinical, decisions. A good prognostic tool can help physicians to tailor treatment to people’s specific needs. The established treatment modality for patients with brain metastases includes tumor surgery. Maximal tumor resection has been proven to be a good prognostic factor. However, widely used prognostic models have not been tested in patients who have undergone surgery. Moreover, the extent of surgery is not incorporated in any prognostic tool. We tested a well-known Graded Prognostic Assessment score and added the rest tumor volume as an additional prognostic factor. The new score provides a good and reliable assessment of prognosis and could be used for further management after surgical treatment.

**Abstract:**

Background: Graded Prognostic Assessment (GPA) has been proposed for various brain metastases (BMs) tailored to the primary histology and molecular profiles. However, it does not consider whether patients have been operated on or not and does not include surgical outcomes as prognostic factors. The residual tumor burden (RTB) is a strong predictor of overall survival. We validated the GPA score and introduced “volumetric GPA” in the largest cohort of operated patients and further explored the role of RTB as an additional prognostic factor. Methods: A total of 630 patients with BMs between 2007 and 2020 were included. The four GPA components were analyzed. The validity of the original score was assessed using Cox regression, and a modified index incorporating RTB was developed by comparing the accuracy, sensitivity, specificity, F1-score, and AUC parameters. Results: GPA categories showed an association with survival: age (*p* < 0.001, hazard ratio (HR) 2.9, 95% confidence interval (CI) 2.5–3.3), Karnofsky performance status (KPS) (*p* < 0.001, HR 1.3, 95% CI 1.2–1.5), number of BMs (*p* = 0.019, HR 1.4, 95% CI 1.1–1.8), and the presence of extracranial manifestation (*p* < 0.001, HR 3, 95% CI 1.6–2.5). The median survival for GPA 0–1 was 4 months; for GPA 1.5–2, it was 12 months; for GPA 2.5–3, it was 21 months; and for GPA 3.5–4, it was 38 months (*p* < 0.001). RTB was identified as an independent prognostic factor. A cut-off of 2 cm^3^ was used for further analysis, which showed a median survival of 6 months (95% CI 4–8) vs. 13 months (95% CI 11–14, *p* < 0.001) for patients with RTB > 2 cm^3^ and <2 cm^3^, respectively. RTB was added as an additional component for a modified volumetric GPA score. The survival rates with the modified GPA score were: GPA 0–1: 4 months, GPA 1.5–2: 7 months, GPA 2.5–3: 18 months, and GPA 3.5–4: 34 months. Both scores showed good stratification, with the new score showed a trend towards better discrimination in patients with more favorable prognoses. Conclusion: The prognostic value of the original GPA was confirmed in our cohort of patients who underwent surgery for BM. The RTB was identified as a parameter of high prognostic significance and was incorporated into an updated “volumetric GPA”. This score provides a novel tool for prognosis and clinical decision making in patients undergoing surgery. This method may be useful for stratification and patient selection for further treatment and in future clinical trials.

## 1. Introduction

The incidence of brain metastases (BMs) has increased owing to several factors, including demographic changes and the increased life expectancy of patients with cancer. They occur in 20–30% of patients with systemic cancer and represent the most common brain tumors, with recurrence rates of approximately 40–60% [1]. Due to the heterogeneous nature of oncologic conditions, the available literature on brain metastasis is still in its infancy.

BM management has evolved secondarily to several factors, including advances in imaging modalities and treatments [2,3,4,5,6], as well as the development of prognostic indices. Several research groups have proposed evaluating different risk factors in patients with cancer to calculate indices to guide treatment decisions [7,8,9,10]. The Graded Prognostic Assessment (GPA) index is a well-known prognostic instrument that assesses the number of BMs, age, Karnofsky performance status (KPS), and extracranial manifestations, making it a valuable tool for assessing treatment outcomes and guiding clinical decisions [11]. Subsequent studies have validated the GPA and demonstrated its utility in clinical practice and trial design. The GPA has been refined and adapted to specific cancer types, such as breast cancer, leading to the development of disease-specific Graded Prognostic Assessment (ds-GPA) indices [12,13,14,15,16]. These indices have been shown to provide valuable prognostic information, aiding in clinical decision making and the stratification of patients in clinical trials [17,18]. For instance, in breast cancer patients with brain metastases, studies have demonstrated that the tumor subtype, such as HER2 and ER/PR status, significantly affects survival [19]. Additionally, imaging characteristics, including peritumoral edema and diffusion-weighted imaging (DWI) signal intensities, have been found to be associated with prognosis in patients with brain metastases [20]. A comprehensive overview of the current approaches to the management of brain metastases emphasizes the individualized nature of treatment for each patient [21].

Surgical resection provides a survival advantage for patients with a single BM. Moreover, surgical treatment results in lower local recurrence rates and better clinical outcomes [3,22]. Resection has been established as the standard therapy for patients with few BMs [23]. Patchell et al. demonstrated that the combination of surgery and whole-brain radiation therapy (WBRT) was more effective in treating patients with a resectable solitary brain metastasis compared to WBRT alone [3]. The study by Patchell and colleagues, along with other trials, such as those by Vecht, provided evidence in favor of surgery for the treatment of single brain metastases. Specifically, the findings indicated that patients who underwent surgical resection followed by WBRT survived significantly longer and had a lower risk of local recurrence compared to those treated with WBRT alone [22]. The importance of considering surgery, particularly in cases of highly radio-resistant tumors, such as non-small cell lung cancer, has been also highlighted. There are also increasing data supporting surgical resection in patients with multiple BMs [24]. These findings underscore the significant clinical advantage of incorporating surgery into the management of single brain metastases, particularly in improving patient survival and reducing the risk of recurrence [25]. There are also increasing data supporting surgical resection in patients with multiple BMs [26,27,28,29,30].

Previous studies [26,27,28,29] have highlighted the significance of the residual tumor burden (RTB) and the extent of resection as robust indicators of extended overall survival (OS) in patients with brain metastases, irrespective of age or cancer type. Postoperative magnetic resonance imaging (MRI) is utilized to assess the extent of resection as an objective measure of surgical outcomes.

This investigation aims to emphasize the importance of considering RTB in the prognostic assessment of a highly diverse histologic group of patients who have undergone surgery for brain metastases, generating a modified version that evaluates the residual tumor volume for a higher postoperative OS estimation accuracy.

## 2. Materials and Methods

### 2.1. Study Population

A total of 630 patients met the inclusion criteria (histopathological diagnosis of BM, pre- and postoperative MRI, tumor resection apart from brain tumor biopsy, and complete medical records) and were included in the final analysis. This study was conducted between April 2007 and January 2020 at the Technical University of Munich.

Patients’ medical records, including age at diagnosis, sex, tumor localization, number of BMs, date of surgery, pre- and postoperative KPS, pre- and postoperative tumor burden, date of death, and/or date of the last follow-up, were evaluated.

### 2.2. Surgical Procedure and Imaging Analysis

The surgical approach aimed to achieve extensive tumor removal and focused on protecting the eloquent areas of the brain. It was performed using pre- and intraoperative navigation techniques. The decision to perform surgery was based on the mass effect, bleeding, development of new neurological deficits, and uncertainty regarding the nature of the tumor. For a detailed description, refer to previously published data [28,29]. All postoperative T1 MRI sequences with contrast enhancement obtained within 72 h postoperatively were analyzed. The contrast-enhancing tumor volumes were manually segmented and analyzed by experienced faculty members using Origin software (Origin, Brainlab, Version 3.1; Brainlab, AG, Munich, Germany).

### 2.3. Statistics

The primary endpoint was to determine the OS after surgery for BM until the date of death or loss to follow-up. Patients lost to follow-up were excluded. The original GPA score categories were applied to the dataset [11], dividing patients into four main categories with previously established cut-offs of 0–1, 1.5–2, 2.5–3, and 3.5–4, where 4 correlated with the best prognosis [7,18,19]. Multivariate analysis with the Cox proportional hazard regression model was used to assess the association between GPA variables and clinical outcomes. Survival analyses for each individual GPA category and score variable were plotted using the Kaplan–Meier curve. Data were individually compared using log-rank statistics. Statistical significance was set at *p* < 0.05. The Bonferroni correction was applied where appropriate. To develop a modified GPA score, a rest tumor volume cut-off of 2 cm^3^ was selected based on previously published results. A clinically relevant cut-off of 1.78 cm^3^ was identified using maximally selected log-rank statistics [29]. To compare the goodness of fit between the two GPA scores, accuracy, sensitivity, specificity, F1-score, and receiver operating characteristic (ROC) analysis were used. Software packages including GraphPad Prism Ver 8.3.1 (La Jolla, CA, USA), SPSS Statistic Ver 29 (IBM Co., Armonk, NY, USA), and MATLAB Ver R2023b were used for analysis. DATAtab eU (Graz, Austria) was used for graphical representation.

## 3. Results

### 3.1. Patient Characteristics

Among the 630 patients that met the inclusion criteria, 350 (50.0%) were male, with a median age at surgery of 63 years (range: 18–93). Patients with BM had a median KPS score of 80% (range: 10–100). Regarding the number of intracranial lesions, 144/630 patients (54.6%) had one, 109/630 (17.3%) had two, 133/630 (21.1%) had three, 7/630 (1.1%) had four, and 47/630 (5.9%) had more than four. Regarding BM localization, 470/630 (74.5%) lesions were in the supratentorial region, 156/630 (24.7%) were in the infratentorial region, and 4/630 (0.8%) were present in both regions.

The GPA scores were stratified into four categories, as follows: 186/630 patients (29.5%) had 0–1, 285/630 had (45.8%) 1.5–2, 133/630 had (21.3%) 2.5–3, and 25/630 had (4%) 3.5–4 points.

Complete cytoreduction was achieved in 444/630 (70.5%) patients, with a median preoperative tumor burden of 12.4 cm^3^ (IQR 5.2– 25.8 cm^3^) and a median postoperative tumor volume of 0.14 cm^3^ (IQR 0.0–2.05 cm^3^). Additional demographic data are presented in Table 1.

### 3.2. GPA Score Validation

KPS, age, number of BM, and presence of extracranial metastases determined GPA scores, with improved clinical characteristics associated with higher GPAs. Data were assessed based on the information obtained preoperatively and at diagnosis. Multivariate Cox hazard analysis affirmed the GPA-OS association (Table 2).

**Figure 1:**
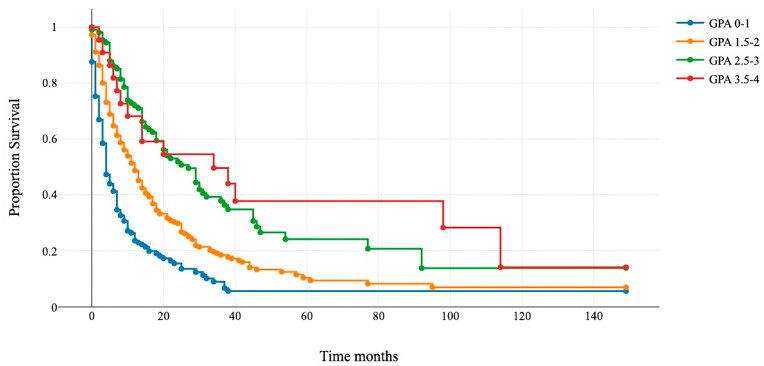
Kaplan–Meier survival curves for the four GPA subgroups.

All patients were assigned to four GPA categories: 0–1, 1.5–2, 2.5–3, and 3.5–4. Kaplan–Meier and log-rank tests showed a significant difference in survival among the four subgroups (Figure 1). The median OS for GPA 0–1 was 4 months (95% confidence interval (CI) 4–5); for GPA 1.5–2, it was 12 months (95% CI 9–13); for GPA 2.5–3, it was 21 months (95% CI 16–29); and for GPA 3.5–4, it was 38 (95% CI 14–114) (Table 3).

Patients that underwent surgery were assigned to four classes: 0–1 (blue line), 1.5–2 (orange line), 2.5–3 (green line), and 3.5–4 (red line). The *x*-axis represents survival after surgery in months, and the *y*-axis shows the percentage of surviving patients. Group comparisons were conducted using the log-rank test (*p* < 0.001).

### 3.3. Rest Tumor Burden as an Independent Predictor for Survival

The RTB independently predicts the OS (hazard ratio (HR) 1.017983, 95% CI 1.0058–1.0303, *p* = 0.0036). Maximally selected log-rank statistics showed a significant RTB cut-off of 1.78 cm^3^ (*p* = 0.0022) for all patients, regardless of the number of intracranial metastases [29]. This value was later rounded to 2 to achieve clarity for its use.

**Figure 2:**
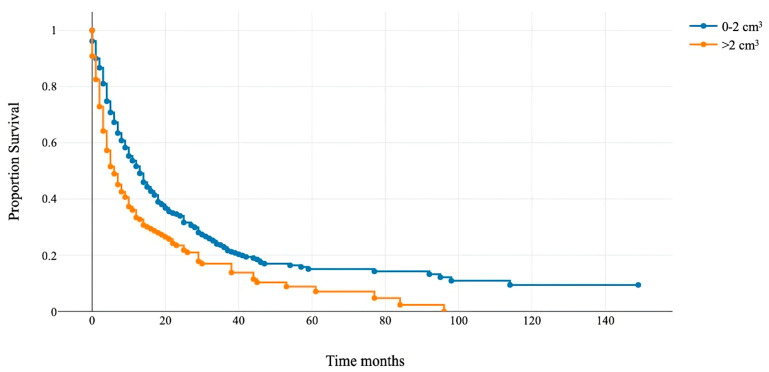
Kaplan–Meier overall survival curve for patients with the rest tumor volume above and below 2 cm^3^.

Patients with RTB > 2 cm^3^ had a median OS of 6 months (95% CI 4–8), and those with RTB < 2 cm^3^ had 13 months (95% CI 11–14) (Figure 2). This cut-off was integrated as an additional variable to modify the GPA score and assess its prognostic accuracy.

The median survival for patients with a rest tumor volume >2 cm^3^ (orange line) was 6 months, and for patients with a rest tumor volume of <2 cm^3^ (blue line), it was 13 months (*p* < 0.001).

### 3.4. Modified GPA Score

To develop a new GPA score, the residual tumor volume was integrated as a fifth category. The age of patients was categorized into two groups: ≤70 years (0.5 points) and ≥70 years (0 points), as we previously reported that age is an independent prognostic factor with a significant cut-off of 67 years [29]. A tumor rest volume ≥2 cm^3^ was given a score of 0, while <2 cm^3^ was assigned a score of 0.5. These values were further divided into four subgroups. Each patient in each subgroup was assigned a score of 0–1, 1.5–2, 2.5–3, and 3.5–4; thus, the values remained comparable with those present in the original GPA. The corresponding scores are listed in Table 4.

**Figure 3:**
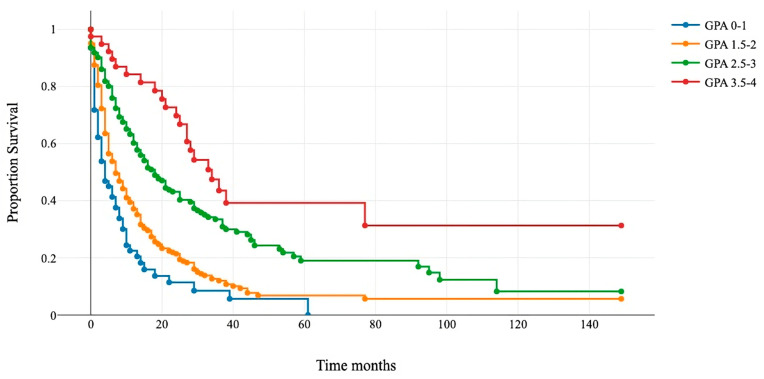
Kaplan–Meier survival curves for the four modified GPA subgroups.

The Kaplan–Meier and log-rank tests demonstrated significant differences in survival among the four categories. The median survival for GPA 0–1 was 4 months; for GPA 1.5–2, it was 7 months; for GPA 2.5–3, it was 18 months; and for GPA 3.5–4, it was 34 months (Figure 3 and Table 5).

Patients that underwent surgery were assigned to four new classes: 0–1 (blue line), 1.5–2 (orange line), 2.5–3 (green line), and 3.5–4 (red line). The *x*-axis represents survival after surgery in months, and the *y*-axis shows the percentage of surviving patients. Group comparisons were conducted using a log-rank test (*p* < 0.01).

The GPA categories were assessed for early death (<3 months) and long-term survival (>12 months), following established methods [31,32]. To compare the classification effectiveness of the standard and modified GPA scores, the accuracy, sensitivity, specificity, F1-score, and ROC AUC (Table 6) were reported.

First, time-dependent specificities filtering patients with short life expectancies (<3 months) were tested. Both scores had similar accuracies (65.4% versus 63.0%), with the sensitivity of the standard score at 43.2% yielding better results. Notably, both scores identified patients with a more favorable prognosis, with “volumetric GPA” showing a distinctively higher specificity (93.0% versus 80.6%). This finding is supported by the F1 scores, the harmonic means of sensitivity, and recall (0.254 and 0.117 for the standard and modified GPA scores, respectively). When the AUC were compared, both scores showed almost identical results.

Thereafter, the measurements of long-term survival (>12 months) were compared. The accuracies increased for both variants (76.5% versus 78.4%), with the modified GPA score marginally outperforming the standard GPA. Comparing the AUC values, the modified GPA also showed better results (0.57 versus 0.53).

There were no significant differences between the two GPA scores. The modified GPA score showed a similar discrimination when compared to the standard GPA; however, it was better at identifying long-term survivors.

## 4. Discussion

BM afflicts 10% of patients with cancer [1,33], with more than 50% presenting with multiple intracranial lesions [34]. These metastases considerably contribute to mortality, morbidity, and healthcare costs [35]. The estimated survival is typically <6 months, yet it greatly varies in a heterogeneous population of these patients [36] and has significantly improved recently [37]. However, surgically treated patients demonstrate a better-than-expected OS [38,39,40], highlighting the need for new prognostic tools.

Surgical resection is a well-established treatment modality for the management of BM [41], and the presence of multiple intracranial lesions is not a contraindication for surgical treatment. Several authors have demonstrated the beneficial role of surgery for solitary and multiple lesions [3,22,42]. In addition to improved survival, neurological benefits can also be achieved with surgical treatment [43]. According to the current guidelines, unknown histology, a single lesion, and symptomatic BM are evaluated as indications for surgical resection [2,30]. Despite the presence of a large cohort of patients who undergo surgical treatment for BM, sufficient data on prognostic evaluation after therapy are lacking. Several attempts have been made to evaluate the clinical outcomes and survival of patients who undergo surgical treatment, with a focus on the extent of resection [44]. However, estimating prognosis after surgery remains challenging.

The original well-established GPA score was developed for patients with their first diagnosis of BM [11]. This score has been validated by multiple studies comparing different disease pathologies without considering further surgical treatment and outcomes [14,18,19,45]. Most studies have focused on stratifying patients based on specific histology, lacking a standardized assessment of disease response to emergent surgical treatment. The GPA and its original scoring items were selected for our analyses because it is one of the most well-established and widely validated prognostic indices for BM.

Some studies have focused on the validation of the GPA in patients with single or multiple metastases. Nieder et al. demonstrated the validity of the GPA in 64 patients who underwent surgery followed by whole-brain radiotherapy [46]. Jakola et al. reported similar results in a cohort of 141 patients [47]. Grossenbacher et al. confirmed the prognostic value of the GPA with 285 patients who underwent surgical treatment for BM [48].

In this study, the prognostic value of the GPA was assessed in the largest cohort of patients who underwent BMs. Based on multivariate Cox analysis, each single score item of the GPA was associated with patient survival, with age and the presence of extracranial lesions having the strongest predictive value. Categorization into four groups depicted a strong contrast in survival, making it possible to assess prognosis after surgical treatment. The current study confirmed the prognostic value of all the components of the original GPA in individuals that underwent surgery.

The aim of this study was to validate the existing score and to develop a modified GPA incorporating the residual tumor volume. The postoperative residual tumor volume has a strong prognostic value for survival and clinical outcomes [28,29,48]. Maximum cytoreduction suggests improved patient survival after surgery [28,29,49,50]. However, notably, some authors did not find an association between the extent of resection and clinical outcomes [42]. Our study group emphasizes that the extent of resection and residual tumor volume should remain integral to prognostic assessment.

The results of the previously published postoperative rest volumes were used in this study [29]. One group was defined as patients with a residual volume >2 cm^3^, and another group as those with a residual volume <2 cm^3^. A significant difference in survival was observed between the two groups; therefore, this division was used for GPA modification. We added a new volumetric parameter to the original prognostic index as one of the objective characteristics of surgical intervention and changed the age categorization to maintain the score within the standard grouping.

Both scores successfully discriminated OS. Both the original and “volumetric GPA” were associated with OS in our analysis, showing similar median survival values. However, the data analysis from this study suggests that the modified GPA with integrated tumor residual volume can better classify long-term survivors. As mentioned previously, a trend towards improved survival was observed in heterogeneous groups of patients with BM, with a mean OS > 10 months in those who received surgical treatment [51,52]. This tendency warrants the development of new evaluation models that can predict prognosis with improved accuracy. The latter emphasizes that maximal tumor resection, tumor volumetric analysis, and a postoperative MRI are essential for evaluating the prognosis of patients with BM. The addition of RTB increased the discriminatory power of the score and selected patients with particularly favorable prognoses.

Other modified GPA scores have been created since the introduction of the standard GPA in 2008 [53], employing several factors specific to the histologic origin of the primary disease, as well as other properties and predictors. These scales include the Diagnosis-specific Graded Prognostic Assessment (DS-GPA), Extracranial Score (EC-S), Updated Renal GPA, Updated Gastrointestinal GPA, Integrated Melanoma DS-GPA, Melanoma Mol-GPA, Sarcoma GPA, Hepatocellular Carcinoma GPA (HCC-GPA), Colorectal Cancer GPA (CRC-GPA), and Uterine Cancer GPA (Uterine GPA). The primary tumor origin was not specified in the standard GPA and the EC-S, while all the others included disease-specific predictors such as the time of primary diagnosis, BRAF gene status, Child–Pugh score, tumor markers such as serum CEA, and neurologic symptoms. The DS-GPA was the more comprehensive one in this cohort of scales, since it evaluated the specific histologic type and included it in the score. In a systematic review performed by Marques-Ribeiro and collaborators [53], they reported that GPA-derived diagnosis-specific scales were superior to the original score. It appears that age and the KPS have frequently been accounted for in previous scores, but the tumor rest volume [28,29,50] has not been considered previously, much less as part of a comprehensive score. We strongly believe that an integrated approach to all BM, regardless of the histologic type, should include surgical resection, whenever it is possible. The latter should always aim for gross total resection, and the tumor rest volume should be assessed along with the GPA in order to make better-informed decisions in the management of patients with BM; hence the necessity to create a score that could be utilized in clinical practice.

Despite the high heterogeneity, poor prognosis, and various therapeutic modalities of BM, surgery is crucial in these patients. A better understanding of prognosis is vital for personalized clinical management. In the largest reported series of patients treated for BM, the understanding of prognosis improved, and a new prognostic factor was identified. The set of selected values incorporated into the “volumetric GPA” index aids in assessing patient prognosis post surgery. The updated version provides a more accurate survival estimation score. These results could inform clinical decisions and standardize the evaluation of a highly diverse group of patients receiving surgical treatment.

The current study had limitations. First, it was a retrospective, single-center design, leading to potential bias due to the loss of follow-up, incomplete medical records, and selection bias. Second, heterogeneity in tumor histology, anatomical localization, and treatment modalities could further complicate data interpretation. Lastly, our study focused exclusively on surgically treated patients with BM, which may have led to a bias towards fitter individuals.

## 5. Conclusions

Emerging evidence indicates that surgical resection plays a primary role in patients with BM. A good prognostic tool is essential in clinical practice and decision making. GPA has become one of the most widely used prognostic scores. However, it does not focus on the subgroup of operated patients. In the current study, the prognostic value of the original GPA has been confirmed in numerous patients who have undergone surgery. The tumor rest volume has been shown to have prognostic significance for overall survival. Nevertheless, it is not incorporated into the currently used prognostic assessments. The “Volumetric GPA” facilitates the prognosis of individuals after neurosurgical treatment, integrating the tumor rest volume and allowing for a reliable assessment of the prognosis, and could be used for further evaluation after performing surgical treatment. It is essential in perioperative assessment to evaluate the possibility of achieving maximal resection to harness long-term benefits; thus, this score is useful for identifying the patients with the best prognosis, confirming the importance of maximal tumor resection and postoperative volumetric analysis in patients with BM. In summary, the modified index integrates four simple parameters and provides a direction for clinical management.

## Figures and Tables

**Table 1:** Patient characteristics.

Characteristic	Value	Characteristic	Value
Age median (years)	63 (18–93)	Postoperative GPA (score, %)	
(*n*, %)		0–1	186 (29.5)
<45	65	1.5–2	1.5–2
45–55	135	2.5–3	2.5–3
56–60	155	3.5–4	3.5–4
Gender (*n*, %)		Chemotherapy for BM (*n*, %)	
Male	315 (50)	Yes	301 (47.8)
Female	315 (50)	No	289 (45.8)
KPS (*n*, %)	Median 80	Unknown	40 (6.4)
90–100	241 (38.3)	Radiotherapy (*n*, %)	505 (71.7)
70–80	264 (42)	WBRT (*n*, %)	
50–60	84 (13.3)	Yes	208 (41.2)
>40	41 (6.4)	No	297 (58.8)
ECOG (*n*, %)		SRS	
0	39 (6.2)	Yes	26 (5.2)
1	329 (52.2)	No	479 (94.8)
2	162 (25.7)	HSRS	
3	42 (6.7)	Yes	231 (45.7)
4	25 (4)	No	274 (54.2)
Unknown	33 (5.2)	Complete cytoreduction (*n*, %)	
Histology (*n*, %)		Yes	444 (70.5)
Lung cancer	128 (20.3)	No	186 (29.5)
Melanoma	107 (17)	BM localization (*n*, %)	
Breast cancer	100 (15.9)	Supra-tentorial	470 (74.5)
CRC	45 (7.1)	Infra-tentorial	156 (24.7)
RCC	35 (5.6)	Both	4 (0.8)
Prostata	23 (3.7)	Tumor volume (cm^3^, median IQR)	
CUP	19 (3)	Preoperative	12.4 cm^3^ (5.2–25.8 cm^3^)
Others	173 (27.4)	Postoperative	0.14 cm^3^ (0.0–2.05 cm^3^)
Number of lesions (*n*, %)			
1	344 (54.6)		
2	109 (17.3)		
3	133 (21.1)		
4	7 (1.1)		
>4	37 (5.9)		

(KPS) Karnofsky performance scale, (ECOG) Eastern Cooperative Oncology Group status, (CRC) colorectal cancer, (RCC) renal cell carcinoma, (CUP) cancer of unknown primary, (BM) brain metastasis, (IQR) interquartile range, (GPA) graded prognostic assessment, (SRS) stereotactic radiosurgery, (HSRS) hypofractionated stereotactic radiotherapy.

**Table 2:** Multivariate COX hazard analysis of GPA score categories applied to our patient cohort.

Category	*p* Value	HR	Lower 95% Cl	Upper 95% Cl
Age (<50 vs. 50–60 vs. >60)	<0.001	2.89	2.51	3.32
KPS (80–100 vs. 60–70 vs. ≤50)	<0.001	1.32	1.16	1.51
Number of BMs (1 vs. 2–3 vs. ≥4)	0.019	1.38	1.05	1.81
Extracranial BMs (no, yes)	<0.001	2.03	1.62	2.55

First column represents the GPA score items, second column represents two-sided *p*-values, third column represents hazard rations (HRs), and the last two columns states the 95% confidence intervals (CIs). KPS: Karnofsky performance score, BM: brain metastasis. *p* < 0.05 was considered significant.

**Table 3:** Overall survival of four GPA classes showed in months.

	Mean Estimate	Median Estimate	Median Lower 95% CI	Median Upper 95% CI
GPA 0–1	9.66	4	4	5
GPA 1.5–2	22.17	12	9	13
GPA 2.5–3	34.73	21	16	29
GPA 3.5–4	57.9	38	14	114

**Table 4:** Modified GPA Scores with integrated tumor rest volumes.

	0	0.5	1
Age	≥70	<70	NA
KPS	<70	70–80	90–100
ECM	no	NA	yes
N of BM	>3	2–3	1
Rest tumor volume	≥2	<2	NA

(KPS) Karnofsky performance scale, (ECM) extracranial manifestation, (BM) brain metastasis, (NA) not applicable.

**Table 5:** Overall survival of the four modified GPA classes shown in months.

	Mean Estimate	Median Estimate	Median Lower 95% CI	Median Upper 95% CI
GPA 0–1	10.16	4	3	6
GPA 1.5–2	15.69	7	6	9
GPA 2.5–3	35.08	18	14	23
GPA 3.5–4	43.38	34	27	77

**Table 6:** Comparison of the classification performance for 3- and 12-month survival.

	Short-Term Survival		Long-Term Survival	
Measures	Standard GPA Score	Modified GPA Score	Standard GPA Score	Modified GPA Score
Accuracy	65.4%	63%	76.5%	78.4%
Sensitivity	43.2%	20.1%	8.2%	17.1%
Specificity	80.6%	93.0%	97.1%	97.0%
F1 score	0.254	0.117	0.031	0.064
AUC	0.62	0.6	0.53	0.57

Comparison of the two GPA scores for 3-month survival, showing both scores performing equally (McNemar test, *p* = 0.31). The comparison of the classification of the 12-month and longer survival category showed similar results. There was no statistically significant difference in performance (McNemar test, *p* = 0.18).

## Data Availability

The datasets generated during and/or analyzed during the current study are available from the corresponding author on reasonable request.
